# Drug-related problems and associated factors in Ethiopia: a systematic review and meta-analysis

**DOI:** 10.1186/s40545-021-00312-z

**Published:** 2021-04-26

**Authors:** Fuad Adem, Jemal Abdela, Dumessa Edessa, Bisrat Hagos, Abraham Nigussie, Mohammed A. Mohammed

**Affiliations:** 1 Department of Clinical Pharmacy, School of Pharmacy, College of Health and Medical Sciences, Haramaya University, Harar, Ethiopia; 2 Department of Pharmacology and Toxicology, School of Pharmacy, College of Health and Medical Sciences, Haramaya University, Harar, Ethiopia; 3 Department of Pharmaceutical Supply Chain Management, College of Health and Medical Sciences, Haramaya University, Harar, Ethiopia; 4 School of Pharmacy, University of Auckland, Auckland, New Zealand

**Keywords:** Drug-Related problems, Systematic review and meta-analysis, Ethiopia

## Abstract

**Background:**

Drug-related problems (DRPs) can occur at any stages of medication use processes, and a single drug could be associated with multiple DRPs. Once happened, it adversely affects health outcomes. In Ethiopia, evaluation of the magnitude and factors associated with DRPs had not been attempted at the national level.

**Method:**

The literature search was conducted in the following databases; PubMed, Embase, Medline, and Google Scholar. The quality of the included studies was checked using Joanna Brigg’s Institute (JBI’s) checklist, and data were analyzed using Stata software (version 14.0). The pooled estimate of DRPs was computed by a Random effect model (DerSimonian–Laird method). Cochran’s Q test (I^2^) statistic)), and Begg’s correlation and Egger’s regression test were assessed for heterogeneity and publication bias, respectively.

**Result:**

Overall, 32 studies with a total sample size of 7,129 were included in the review. The estimated pooled prevalence of DRPs was 70% [0.70 (95% CI 0.64—0.76; *I*^2^ = 97.6% *p* = 0.000)]. Polypharmacy (taking ≥ 5 drugs) [RR = 1.3], medical comorbidity [RR = 1.3], poor medication adherence [RR = 1.7], uncontrolled blood pressure [RR = 1.4], substance use [RR = 1.2], type 2 diabetes [RR = 1.8], significant drug interaction [RR = 1.33], and a negative medication belief [RR = 3.72] significantly influenced the occurrence of DRPs.

**Conclusion:**

The estimated national prevalence of DRPs in Ethiopia was high**.** Presence of medical comorbidity, using multiple drugs, significant drug interaction, poor medication adherence, uncontrolled blood pressure, type 2 diabetes, substance use and a negative belief about medication significantly influenced the occurrence of DRPs. Initiating and/or strengthening pharmaceutical care services at the health care facilities could lower the occurrence of DRPs. PROSPERO registration number CRD42020162329.

**Supplementary Information:**

The online version contains supplementary material available at 10.1186/s40545-021-00312-z

## Background

Over the past few decades, the role of the pharmacist has evolved from a compounder and supplier of a drug product to a new paradigm of patient-oriented care [1]. This include patient-centered care such as patient counseling, providing drug information, monitoring drug therapy, and supply chain management [1]. Clinical pharmacy is an area of pharmacy practice that combines the science and practice of rationale use of medications [2]. It is more oriented to the analyses of population needs with regards to medicines, ways of administration, patterns of use, and drug effects on patient outcomes [1]. The clinical pharmacy service is a patient-centered service that promotes an appropriate selection and utilization of medications intending to optimize therapeutic outcomes [3].

The practice of clinical pharmacy embraces the philosophy of pharmaceutical care (PC) and is a basic component in delivering and improving the quality of PC [4]. The PC is a patient-centered, outcomes-oriented pharmacy practice that requires the pharmacist to work in concert with the patient and the patient's other health care providers to promote health, prevent disease, and assess, monitor, initiate, and modify medication use to assure that drug therapy regimens are safe and effective [5 ]. The primary goal of PC is to maintain the patient’s quality of life and improve clinical outcomes [5, 6]. It is implemented/practiced through the PC cycle that involves patient assessment, developing a care plan, implementing a care plan, and monitoring and reviewing the care plan [6, 7].

Patient taking medication/s on a regular basis often have some unmet needs with regards to their drug therapy. This may be related to indication, effectiveness, safety, or compliance issues. If these patient drug-related needs are not addressed addressed, the drug treatment may result in undesirable effects [6]. In practicing pharmaceutical care, the clinical pharmacist assesses if the patient's drug-related needs were met and identifies the occurrence of drug-related problems (DRPs) [6, 7]. DRPs are an undesirable event or risk of an event that involves or suspected to involve drug therapy, and it interfere with achieving the desired goal of therapy if happened [6]. The undesirable event can be a medical compliant, sign, symptoms, diagnosis, disease, illness, impairment, disability, abnormal laboratory values, or syndrome [6]. DRPs can be classified into seven categories (unnecessary drug therapy, need additional drug therapy, ineffective drug, low dose, adverse drug reaction, high dose, and non-compliance); and once happened, it negatively interfere with a patients’ health outcomes [6].

Nowadays, the number of drugs in the market have dramatically increased, posing a significant challenge in controlling the safe and rational drug use [1]. This is could be one factor contributing to the occurrence of one or more DRPs. Globally, various studies have been conducted to identify the magnitude and types of DRPs. In one study, every hospitalized patient had one or more DRPs [8]. In a Norwegian systematic review, the prevalence of DRPs ranges from 70% to less than 30% [9]. In a study performed in Malaysia, 90.5% of study subjects had one or more DRPs [10]. Insufficient awareness of health and disease (26%), choice problems (23%), dosing problems (16%), and drug interaction (16%), were the most common DRPs in the study participants DRPs [10]. Based on the study conducted in Minnesota, 70% of participants had one or more DRPs [11]. In a study conducted to identify the magnitude of DRPs, more than half (53.4%) of study subjects had one or more DRPs in which dosing problems (42.7%), selection problems(23.3%) and adverse drug reactions(13.4%) were the commonly identified DRPs [13]. In another study, about one-third (33%) of study subjects had one or more DRPs [14]. The commonly identified DRPs were non-adherence to clinical practice guidelines (29.5%), improper administration (19.6%), drug interaction (16.7%), and high dose (12.8%) [14]. Another study by Koh et al. reported need additional drug therapy (31.3%), non-compliance (28.1%), adverse drug reaction(25%), low dose (12.5%) and high dose (3.1%) were the most commonly encountered DRPs [15].

The occurrence of DRPs could be influenced by different factors including, the number of drugs (taking ≥ 5) and types of medical conditions [13]. In a study by Urbina et al., polypharmacy, female sex and first time admission to the hospital were predictors of DRPs [12]. The perception that one could stop the medication when the condition is under control, and expectation of cure, also contribute to the occurrence of DRPs [16]. Furthermore, the number of prescription drugs, and the number of over-the counter drugs are also common contributing factors to the occurrence of the DRPs [17].

Once occurred, DRPs can cause significant morbidity and mortality and result in an enormous economic burden. In one study, 3.3% of total admission was due to DRPs [17]. In a study performed to assess the morbidity and mortality from DRPs, the total cost of drug-related morbidity and mortality was estimated to be more than $117.4 billion [18]. Furthermore, drug-related morbidity and mortality in chronic care were estimated to be $76.7 billion [19].

Identification and resolution, and prevention of the DRPs is the unique contribution of PC practitioners [6]. To minimize and/or prevent the occurrence of DRPs, and consequent morbidity, mortality, and economic burden of the DRPs, Incorporating and implementation of PC in the health care systems plays a vital role. In a study done to explore the clinical and economic impacts of pharmaceutical care, 61% of DRPs were identified, and upon resolving the problems, it resulted in, 83% improvement in patients’ clinical status and $1,134,162 health care saving [20].

In Ethiopia, the ward-based clinical pharmacy service was introduced in 2013 [21]. Since then, several studies have been conducted to assess the magnitude, types or factors contributed to the occurrence of DRPs. However, data on the DRPs and contributing factors has not been summarized from these studies to get an insight into the magnitude of the problem. Thus, this systematic review and meta-analysis aimed to determine the magnitude of DRPs and associated factors in Ethiopia.

## Method

The Preferred Reporting Items for Systematic review and meta-analyses Protocols (PRISMA) guideline was used in reporting the data. The protocol was registered in the International Register of Systematic Review (PROSPERO) with a registration number of CRD42020162329.

### Data sources and searches

We searched for studies that assessed the prevalence, types and/or factors associated with DRPs as a primary or secondary outcome using keywords (drug-related problems, DRPs, drug therapy problems, DTP, unnecessary drug therapy, ineffective drugs, low dose, high dose, adverse drug reaction, and non-compliance) in the following databases; PubMed, Embase, Medline, Google Scholar. The reference lists of the included studies were reviewed to find additional articles. All published and unpublished studies conducted between 2013 and 2019 were included. The literature search was limited to the English language and studies involving human subjects.

### Screening and eligibility

Titles, abstracts, and/or full articles of all retrieved studies were assessed for eligibility. Studies were included if they assessed the prevalence, types, and/or factors associated with DRPs as primary or secondary outcomes in patients with any medical conditions treated in the outpatient or inpatient care settings in Ethiopia.

### Data extraction

Quality of the included studies was evaluated using Joanna Brigg’s Institute (JBI’s)Critical Appraisal Tools [22, 23]. Relevant information from the included studies was extracted using a data extraction format. Information such as authors’ name, year of publication, study design and setting, the department (a clinic or wards) of health care facility, where the study conducted, medical conditions, sample size, number of patients with DRPs, the total number of DRPs, and sub-types of DRPs (unnecessary drug therapy, need additional drug therapy, ineffective drug, low dose, high dose, adverse drug reaction, and noncompliance) were extracted.

### Outcome variables

The proportion of DRPs was the primary outcome variable in this review. The magnitude of DRPs in each included study was estimated from the sample size, was estimated out of the sample size, pooled, and reported as a proportion. Factors associated with the occurrences of DRPs were considered as secondary outcomes in this review.

### Data synthesis and analysis

Stata software version 14.0 was used for data analyses. The pooled proportion of DRPs was estimated using a random-effect model, and factors associated with the occurrence of DRPs were summarised using. Proportions of the sub-types of DRPs were estimated from the total number of events (DRPs). The average number of DRPs per-patient was computed as a ratio of the total number of events (DRPs) to the number of subjects with events (DRPs). Sensitivity analysis was done to see the influence of specific studies on the occurrence of DRPs. Subgroup analyses were performed by the hospital (Hiwot Fana Specialized University Hospital, Jimma University Medical Centre, Dessie Referal Hospital, Tikur Anbessa Specialized Hospital, Wolaita Referal Hospital, Mizan, Bonga and Tepi General Hospital, Madda-Walabu University Goba Referal Hospital, Zewditu Referal Hospital, Ayder Referal Hospital, Gebre Tsadik Shawo General Hospital, Adama Referal Hospital, Ambo General Hospital, Felege Hiwot Referal Hospital, Dilchora Referal Hospital, Gondar University Referal Hospital), a medical condition (hypertension, diabetes, heart failure, cancer, epilepsy, schizophrenia, and mixed (i.e., unspecified cases from medical, surgical and pediatrics ward) and a hospital department (ambulatory care, medical ward, surgical ward, and pediatrics ward) at which the study performed. The heterogeneity of the included studies was assessed using the Cochran's Q test (Chi-squared (I^2^) statistic). The presence of publication bias was checked using Begg’s correlation and Egger’s regression test. A p-value of ≤ 0.05 was considered significant in all cases.

### Definition of terms [6]

Unnecessary drug therapy: the drug therapy is unnecessary, because the patient does not have a clinical indication.

Need additional drug therapy: additional drug therapy is required to treat or prevent a medical condition in the patient.

Ineffective drug: the drug product is not being effective at producing the desired response in the patient.

Low dose: the dose is too low to produce the desired response in the patient.

Adverse drug reaction: the drug is causing an adverse reaction in the patient.

High dose: the dose is too high, resulting in undesirable effects experienced by the patient.

Non-compliance: the patient is not able or willing to take the drug therapy as intended.

## Result

**Fig. 1:**
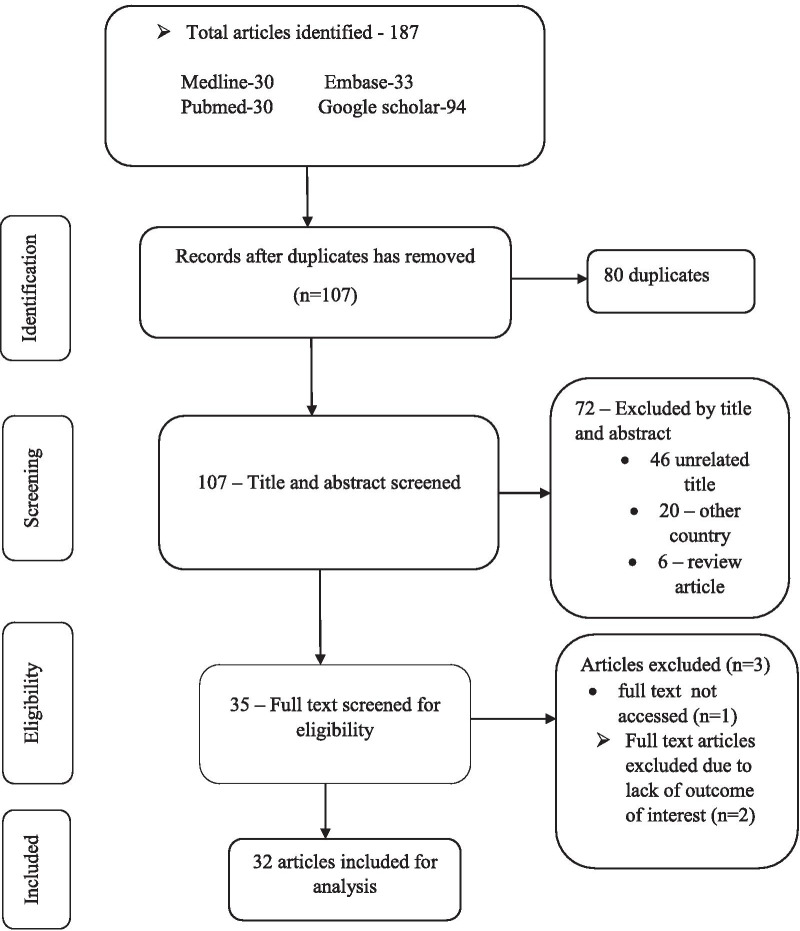
PRISMA flow diagram describing the selection of studies for systematic review and meta-analysis on drug-related problems in Ethiopia

A total of 187 studies related to DRPs were retrieved. Eighty (80) duplicates were removed. The remaining 107 studies were screened by titles and abstracts, and 72 excluded. Of 72 excluded, 46 were not related to the scope of the review, 20 were studies from other countries, and 6 were review articles. Out of the 35 retained studies, the full text was not accessed for one study and thus, it was excluded. The remaining 34 articles full text were reviewed to assess their eligibility for inclusion. Following full text review, two studies were excluded due to, two studies were excluded due to the absence of outcomes of interest. Therefore, 32 studies that met the pre-defined eligibility criteria were included in the review (Fig. 1).

### Characteristics of the study

Overall, 32 studies evaluating the magnitude of DRPs were included in the systematic review and meta-analysis. Studies were conducted among patients with various medical conditions and at different departments within the hospitals such as ambulatory care, medical wards, surgical wards, and pediatrics wards. All included studies assessed the magnitude of DRPs and reported its subtypes (unnecessary drug therapy, need additional drug therapy, ineffective drug, low dose, high dose, and non-compliance). The total sample for the included studies was 7129, ranging from 76 in the study done at Felege Hiwot Referal Hospital [24] to 423 in the study performed at Tikur Anbessa Specialized Hospital [25]. Of the total subjects from included studies, 4764 had one or more DRPs with an estimated 18,956 total number of DRPs (Table 1).

**Table 1:** Quality assessment of the included studies

References	JBI’s critical appraisal checklist, 2017										
	Q1	Q2	Q3	Q4	Q5	Q6	Q7	Q8	Q9	Q10	Q11
Mohammednur et al. 2014 [45]	Yes	Yes	No	yes	No	No	Yes	No	NA	NA	NA
Gebre et al. 2017 [34]	Yes	yes	No	Yes	Yes	Yes	Yes	Yes	NA	NA	NA
Gobezie et al. 2014 [46]	Yes	Yes	No	Yes	No	Yes	Yes	Yes	NA	NA	NA
Gobezie et al. 2013 [24]	Yes	No	No	Yes	Yes	Yes	Yes	UC	Yes	No	Yes
Aster et al. 2019 [26]	Yes	No	No	Yes	Yes	Yes	Yes	UC	Yes	No	Yes
Yaschilal et al. 2014 [43]	Yes	No	No	Yes	Yes	Yes	Yes	UC	Yes	No	NA
Abadir et al. 2015 [47]	Yes	Yes	No	Yes	Yes	Yes	Yes	Yes	NA	NA	NA
Gashaw et al. 2016 [54]	Yes	Yes	No	Yes	Yes	Yes	Yes	Yes	NA	NA	NA
Yirga et al. 2015 [27]	Yes	No	No	Yes	Yes	Yes	Yes	UC	Yes	No	Yes
Tamene et al. 2014 [28]	Yes	No	No	Yes	Yes	Yes	Yes	UC	Yes	No	Yes
Bereket et al. 2014 [29]	Yes	Yes	No	Yes	Yes	Yes	Yes	Yes	NA	NA	NA
Mohammed et al. 2016 [30]	Yes	Yes	No	Yes	Yes	Yes	Yes	Yes	NA	NA	NA
Mequanent et al. 2014 [55]	Yes	Yes	No	Yes	Yes	Yes	Yes	Yes	NA	NA	NA
Asgedom et al. 2016 [48]	Yes	Yes	No	Yes	Yes	Yes	Yes	Yes	NA	NA	NA
Kaleab et al. 2015 [49]	Yes	Yes	No	Yes	Yes	No	Yes	No	NA	NA	NA
Eskinder et al. 2013 [33]	Yes	Yes	No	Yes	Yes	Yes	Yes	Yes	NA	NA	NA
Hailu et al. 2015 [50]	Yes	Yes	No	Yes	Yes	Yes	Yes	Yes	NA	NA	NA
Malede et al. 2018 [36]	Yes	No	No	Yes	Yes	Yes	Yes	UC	Yes	No	Yes
Beshir et al. 2017 [37]	Yes	Yes	No	Yes	Yes	Yes	Yes	Yes	NA	NA	NA
Elham et al. 2017 [25]	Yes	Yes	No	Yes	Yes	Yes	Yes	Yes	NA	NA	NA
Mohammed et al. 2014 [38]	Yes	Yes	No	Yes	Yes	Yes	Yes	Yes	NA	NA	NA
Tadele et al. 2015 [53]	Yes	No	No	Yes	Yes	Yes	Yes	UC	Yes	No	Yes
Ayele et al. 2017 [41]	Yes	Yes	No	Yes	Yes	Yes	Yes	Yes	NA	NA	NA
Tamene et al. 2017 [28]	Yes	Yes	No	Yes	Yes	Yes	Yes	Yes	NA	NA	NA
Haymen et al. 2018 [40]	Yes	Yes	No	Yes	Yes	Yes	Yes	No	NA	NA	NA
Berhane et al. 2017 [31]	Yes	No	No	Yes	Yes	Yes	Yes	UC	Yes	No	Yes
Gobezie et al. 2013 [50]	Yes	Yes	No	Yes	Yes	Yes	Yes	Yes	Yes	No	Yes
Yohannes et al. 2017 [41]	Yes	Yes	No	Yes	Yes	No	Yes	No	NA	NA	NA
Bereket et al. 2013 [32]	Yes	Yes	No	Yes	Yes	Yes	Yes	Yes	NA	NA	NA
Gizachew et al. 2018 [44]	Yes	No	No	Yes	Yes	Yes	Yes	UC	Yes	No	Yes
Gubae et al. 2017 [39]	Yes	Yes	No	Yes	Yes	No	Yes	Yes	NA	NA	NA
Gosaye et al. [33]	Yes	No	No	Yes	Yes	Yes	Yes	UC	Yes	No	Yes

*NA* not applicable, UC-unclear; Q1-11, JBI's Critical Appraisal Checklist for Cohort studies [Q1: Were the two groups similar and recruited from the same population? Q2: Were the exposures measured similarly to assign people to both exposed and unexposed groups? Q3: Was the exposure measured in a valid and reliable way? Q4: Were confounding factors identified? Q5: Were strategies to deal with confounding factors stated? Q6: Were the groups/participants free of the outcome at the start of the study (or at the moment of exposure)? Q7: Were the outcomes measured in a valid and reliable way? Q8: Was the follow-up time reported and sufficient to be long enough for outcomes to occur? Q9: Was follow up complete, and if not, were the reasons to loss to follow up described and explored? Q10: Were strategies to address incomplete follow up utilized? Q11: Was appropriate statistical analysis used?]; Q1-8, JBI's Critical Appraisal Checklist for Analytical Cross-Sectional studies [Q1: Were the criteria for inclusion in the sample clearly defined? Q2: Were the study subjects and the setting described in detail? Q3: Was the exposure measured in a valid and reliable way? Q4: Were objective, standard criteria used for measurement of the condition? Q5: Were confounding factors identified? Q6: Were strategies to deal with confounding factors stated? Q7: Were the outcomes measured in a valid and reliable way? Q8: Was an appropriate statistical analysis used?]

Of the total included studies, eight studies were conducted in Jimma University Medical Center (JUMC) [26–33], seven were conducted in Tikur Anbesa Specialized Hospital [25, 34–39]. Three studies were conducted in Hiwot Fana Specialized University Hospital [40]– [42] and two studies were conducted in Dessie Referral Hospital [43, 44]. Majority (*n* = 20) of the studies were conducted in ambulatory care [24, 25, 27, 30, 34–37, 39–42, 45–52], while eight studies were conducted in the medical wards [26, 28, 29, 32, 38, 43, 49, 53]. Four studies were focused on hypertensive patients [45–48], four studies conducted among patients with diabetes [34, 40, 50, 51] and six studies were conducted among heart failure patients [25, 27, 42, 49, 52, 52]. Furthermore, two studies involved diabetic patients with comorbid hypertension [30, 41] (Table 1).

### Proportion with DRPs

**Fig. 2:**
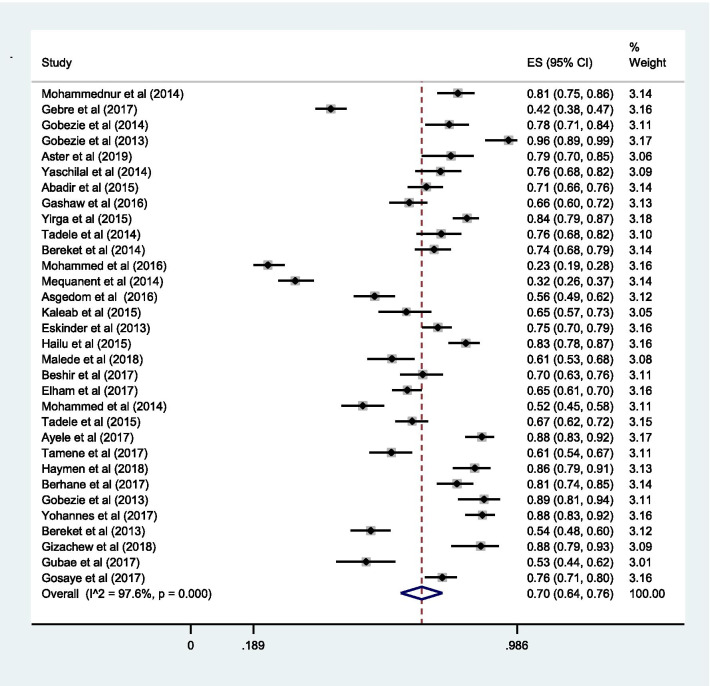
Pooled estimate of the proportion of drug-related problems in Ethiopia

**Fig. 3:**
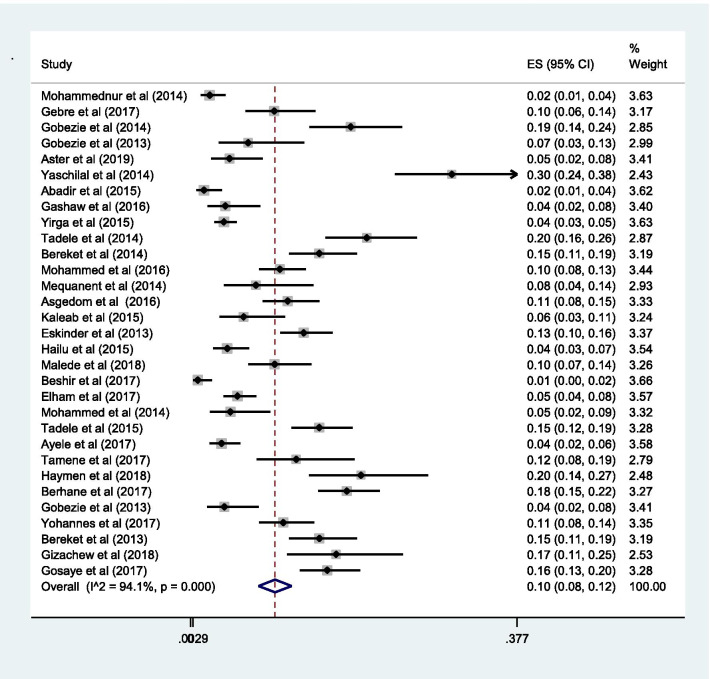
Sub-group analysis of drug-related problems by unnecessary drug therapy in Ethiopia

**Fig. 4:**
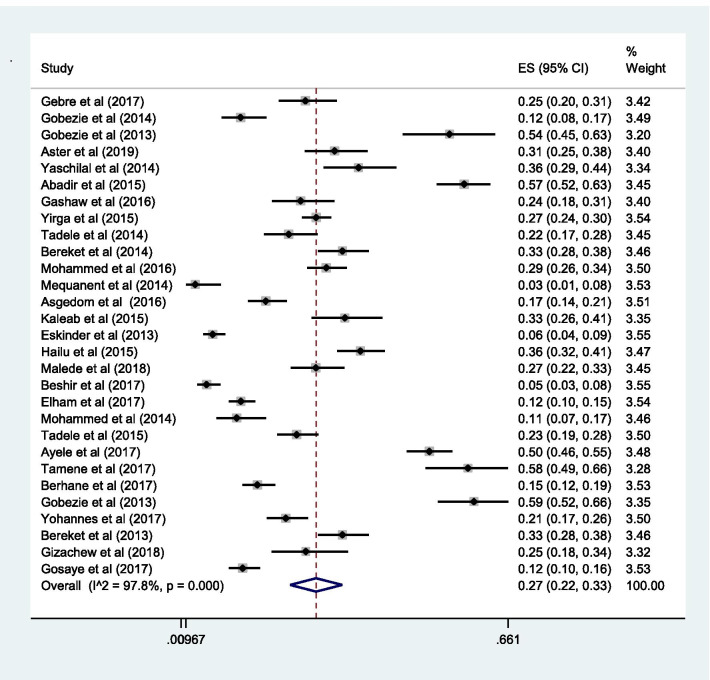
Sub-group analysis of drug-related problems by need additional drug therapy in Ethiopia

**Fig. 5:**
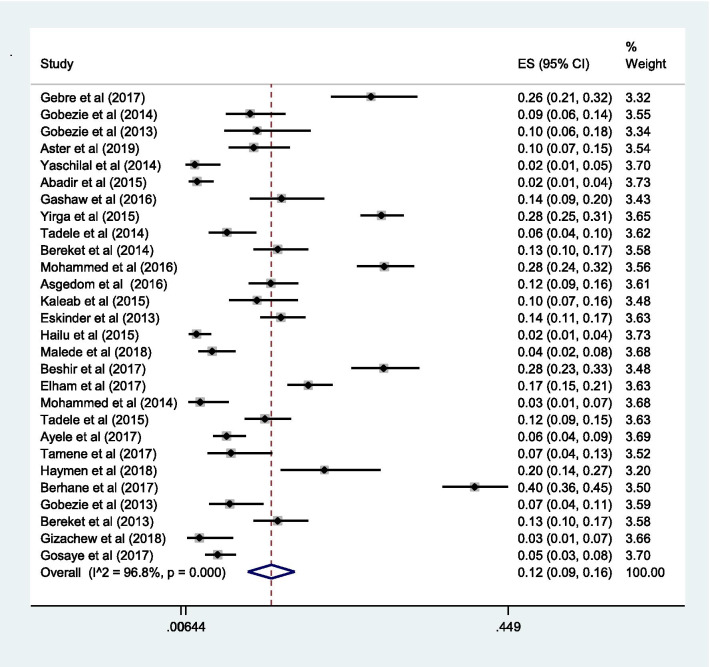
Sub-group analysis of drug-related problems by ineffective drug therapy in Ethiopia

The pooled estimate of the prevalence of DRPs was 70% [0.70 (95% CI 0.64—0.76; *I*^2^ = 97.6% *p* = 0.000)] (Fig. 2). The mean number of DRPs per patient was estimated to be 3.89. Moreover, the pooled estimate of sub-types of DRPs was computed. Accordingly, the pooled estimate of indication related problems (unnecessary drug therapy) [10% (0.10; 95% CI 0.08–0.12; *I*^2^ = 94.1% *p* = 0.000)] (Fig. 3) was lower as compared to the pooled estimate of the other indication related problems (need additional drug therapy) [27%(0.27; 95% CI 0.22–0.33;* I*^2^ = 97.8% *p* = 0.000)] (Fig. 4) and the effectiveness related problems (ineffective drug therapy) [12% (0.12; 95% CI 0.09–0.16;* I*^2^ = 96.8 *p* = 0.000)] (Table 2, Fig. 5).

**Table 2:** Characteristics of the included studies

Author	Year	Design	Population	Study area	Department of the hospital at which study performed	Medical condition	Sample size	Number with Event (DRPs)	Total number of event (DRPs)	Sub-types of drug therapy problems						Sub-types of DRPs
										Unnecessary drug therapy	Need additional drug therapy	Ineffective drug	Low dose	High dose	Adverse drug reaction	Non-compliance
Mohammednur et al [45]	2014	Cross-sectional	Adult	Adama Referal Hospital	Ambulatory care	Hypertension	192	155	452	10			90	81	179	86
Gebre et al [34]	2017	Cross-sectional	Adult	Tikur Anbessa Specialized Hospital	Ambulatory care	Diabetes	418	177	207	20	52	54	58	5	18	
Gobezie et al [46]	2014	Cross-sectional	Adult	Ambo General Hospital	Ambulatory care	Hypertension	151	118	200	37	24	19	24		15	81
Gobezie et al [24]	2013	Prospective general cohort study	Adult	Felege Hiwot Referal Hospital	Ambulatory care	Heart failure	76	73	104		57	11	6	10	20	
Aster et al [26]	2019	Prospective general cohort study	Adult	Jimma University Medical Centre	Medical ward	Chronic kidney disease	103	81	200	9	62	20	36	29	4	40
Yaschilal et al [43]	2014	Prospective general cohort study	Adult	Dessie Referal Hospital	Medical ward	Mixed	147	111	159	48	57	3	21	6	15	9
Abadir et al [47]	2015	Cross-sectional	Adult	Dilchora Referal Hospital	Ambulatory care	Hypertension	271	193	318	5	182	21				124
Gashaw et al [54]	2016	Cross-sectional	Adult	Gondar University Referal Hospital	Medical ward	Mixed	256	169	174	7	42		36	32	8	25
Yirga et al [27]	2015	Prospective observational study	Adult	Jimma University Medical Centre	Ambulatory care	Heart failure	340	284	883	34	241	243	245	14	26	80
Tamene et al [28]	2014	Prospective observational study	Adult	Jimma University Medical Centre	Medical ward	Mixed	152	115	221	45	48	14	48	23	14	29
Bereket et al [29]	2014	Cross-sectional	Adult	Jimma University Medical Centre	Medical ward	Mixed	257	189	316	47	103	42	44	49		31
Mohammed et al [30]	2016	Cross-sectional	Adult	Jimma University Medical Centre	Ambulatory care	Diabetes and Hypertension	300	70	494	51	145	138	78	9	13	60
Mequanent et al [55]	2014	Cross-sectional	Paediatrics	Zewditu Referal Hospital	Paediatrics ward	Mixed	285	90	106	8	3		35	36	24	
Asgedom et al [48]	2016	Cross-sectional	Adult	Ayder Referal Hospital	Ambulatory care	Hypertension	241	134	357	40	61	44	60	3	6	143
Kaleab et al [49]	2015	Cross-sectional	Adult	G/Tsadik Shawo General Hospital	Ambulatory care	Heart failure	132	86	163	10	54	17	4	11	21	46
Eskinder et al [35]	2013	Cross-sectional	Adult	Tikur Anbessa Specialized Hospital	Ambulatory care	Cancer	367	274	474	62	30	65	69	78	170	
Hailu et al [50]	2015	Cross-sectional	Adult	Wolaita Referal Hospital	Ambulatory care	Diabetes	243	202	378	16	137	8	65	14	11	127
Malede et al [36]	2018	Prospective observational study	Paediatrics	Tikur Anbessa Specialized Hospital	Ambulatory care	Cancer	176	107	257	25	70	11	60	41	14	36
Beshir et al [37]	2017	Cross-sectional	Adult	Tikur Anbessa Specialized Hospital	Ambulatory care	Epilepsy	191	134	352	3	18	98	39	33	161	
Elham et al [25]	2017	Cross-sectional	Adult	Tikur Anbessa Specialized Hospital	Ambulatory care	Heart failure	423	277	572	31	69	100	129	82	161	
Mohammed et al [38]	2014	Cross-sectional	Adult	Tikur Anbessa Specialized Hospital	Medical ward	Mixed	225	117	152	7	17	4	11	34	59	
Tadele et al [53]	2015	Prospective observational study	Adult	Mizan, Bonga & Teppi General Hospital	Medical ward	Mixed	348	233	390	58	91	45	65	46	22	63
Ayele et al [41]	2017	Cross-sectional	Adult	Madda-Walabu University Goba Referal Hospital	Ambulatory care	Diabetes	216	190	446	16	224	28	55	3	23	97
Tamene et al [42]	2017	Cross-sectional	Adult	Hiwot Fana Specialized University Hospital	Ambulatory care	Heart failure	216	131	131	16	76	9	14			16
Haymen et al [40]	2018	Cross-sectional	Adult	Hiwot Fana Specialized University Hospital	Ambulatory care	Diabetes	148	127	127	25		25	46			31
Berhane et al [31]	2017	Prospective observational study	Adult	Jimma University Medical Centre	Medical and Surgical ward	Mixed	200	161	449	81	69	181			92	26
Gobezie et al [50]	2013	General cohort study	Adult	Jimma University Medical Centre & Felege Hiwot Referal Hospital	Ambulatory care	Heart failure	97	86	179	7	106	12	5	27	22	
Yohannes et al [41]	2017	Cross-sectional	Adult	Hiwot Fana Specialized University Hospital	Ambulatory care	Diabetes and hypertension	203	179	364	39	77				69	179
Bereket et al [32]	2013	Cross-sectional	Adult	Jimma University Medical Centre	Medical ward	Mixed	257	140	316	47	103	42	44	49		31
Gizachew et al [44]	2018	Prospective observational study	Paediatrics	Dessie Referal Hospital	Paediatrics ward	Mixed	81	71	119	20	30	3	22	10	10	24
Gubae et al [39]	2017	Cross-sectional	Adult	Tikur Anbessa Specialized Hospital	Ambulatory care	Schizophrenias	117	62								
Gosaye et al [33]	2017	Cross-sectional	Adult	Jimma University Medical Centre	Surgical ward	Mixed	300	228	418	66	52	21	124	81	26	48

**Fig. 6:**
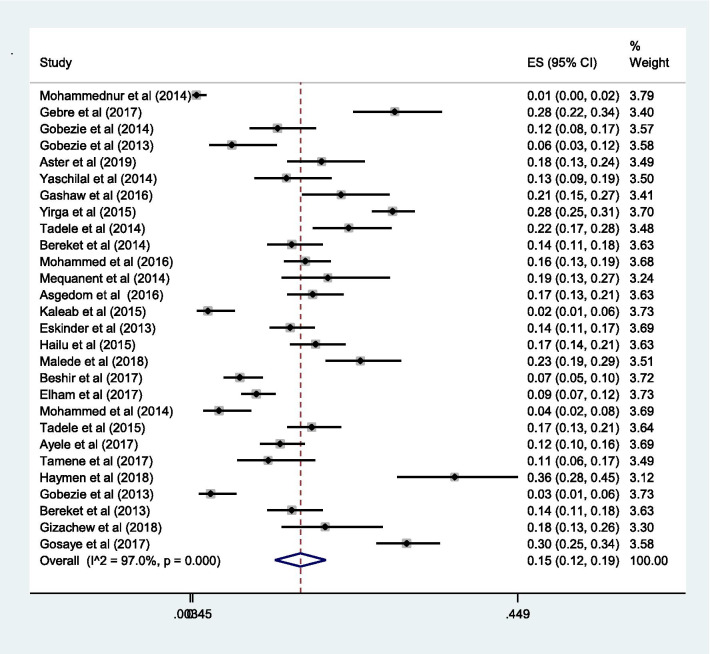
Sub-group analysis of drug-related problems by low dose in Ethiopia

**Fig. 7:**
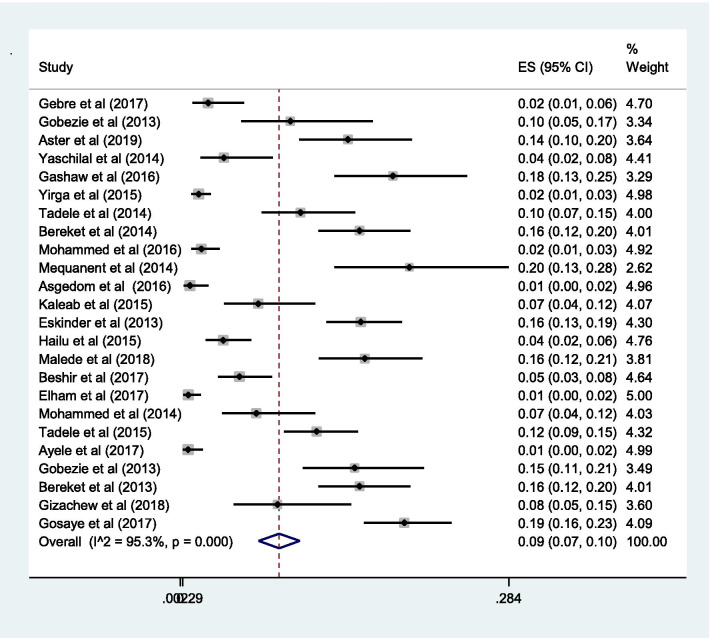
Sub-group analysis of drug-related problems by high dose in Ethiopia

**Fig. 8:**
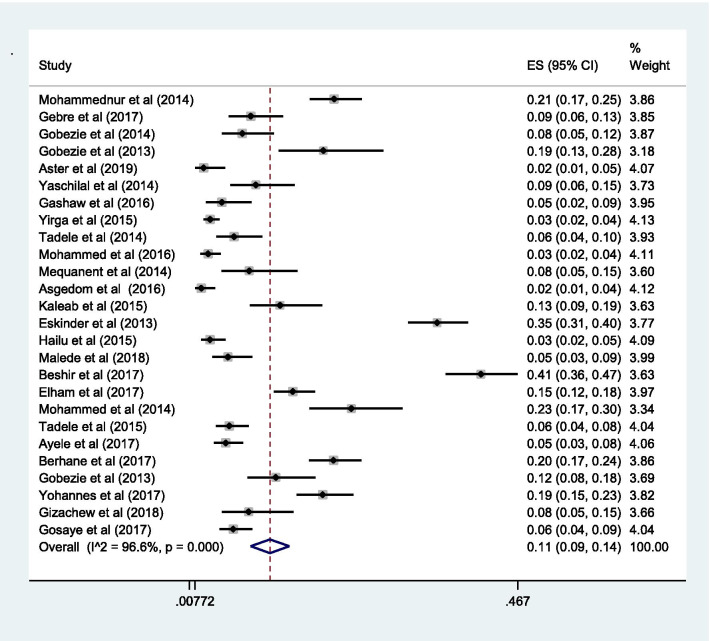
Sub-group analysis of drug-related problems by adverse drug reaction in Ethiopia

**Fig. 9:**
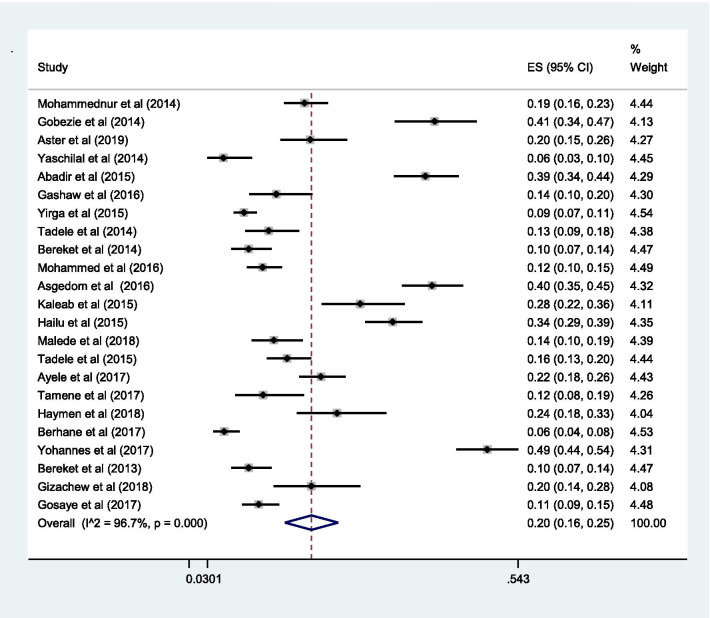
Sub-group analysis of drug-related problems by non-compliance in Ethiopia

The pooled estimate of effectiveness related problems (low dose) [15% (0.15; 95% CI 0.12–0.19;* I*^2^ = 97.0% *p* = 0.000)] (Fig. 6) was higher than the pooled estimate of both the safety related problems; high dose [9% (0.09; 95% CI 0.07–0.10;* I*^2^ = 95.3 *p* = 0.000)] (Fig. 7) and adverse drug reactions [11% (0.11; 95% CI 0.09–0.14;* I*^2^ = 96.6% *p* = 0.000)] (Fig. 8); however, it was lower than the pooled estimate of compliance related problems (non-compliance) [20% (0.20; 95% CI 0.16–0.25;* I*^2^ = 96.7% *p* = 0.000)] (Fig. 9).

### Sensitivity and sub-groups analysis

**Fig. 10:**
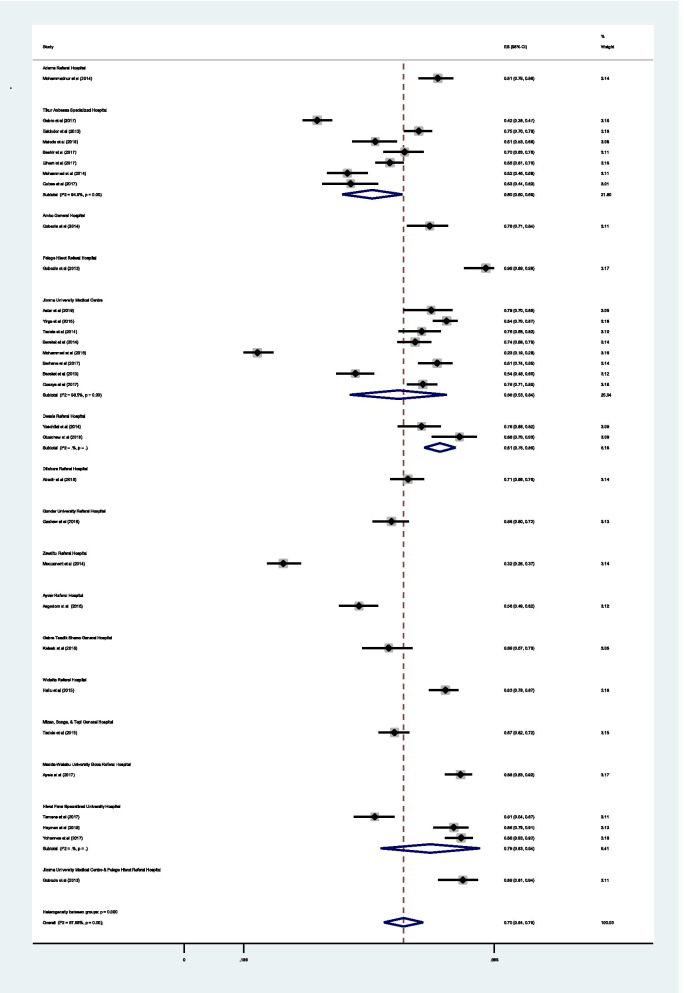
Sub-group analysis of drug-related problems by the hospital in which the study conducted

Sensitivity analysis was performed and a one-on-one removal of studies did not show any effect on the findings (i.e. occurrence of DRPs). Subgroup analysis was done by the hospital, hospital department (Ambulatory care, Medical ward, Pediatrics ward, and surgical ward) and medical conditions (hypertension, diabetes, heart failure, epilepsy, schizophrenia, cancer and mixed (i.e., unspecified cases from medical, surgical and pediatrics ward)). Accordingly, the pooled prevalence of DRPs at Jimma University Medical Centre [68% (0.68; 95% CI 0.53 -0.84; *I*^2^ = 98.48% *p* = 0.00)] was higher compared to the estimated prevalence at Tikur Anbessa Specialized Hospital [60% (0.60; 95%CI 0.50–0.69; *I*^2^ = 98.48% *p* = 0.00)] (Fig. 10).

**Fig. 11:**
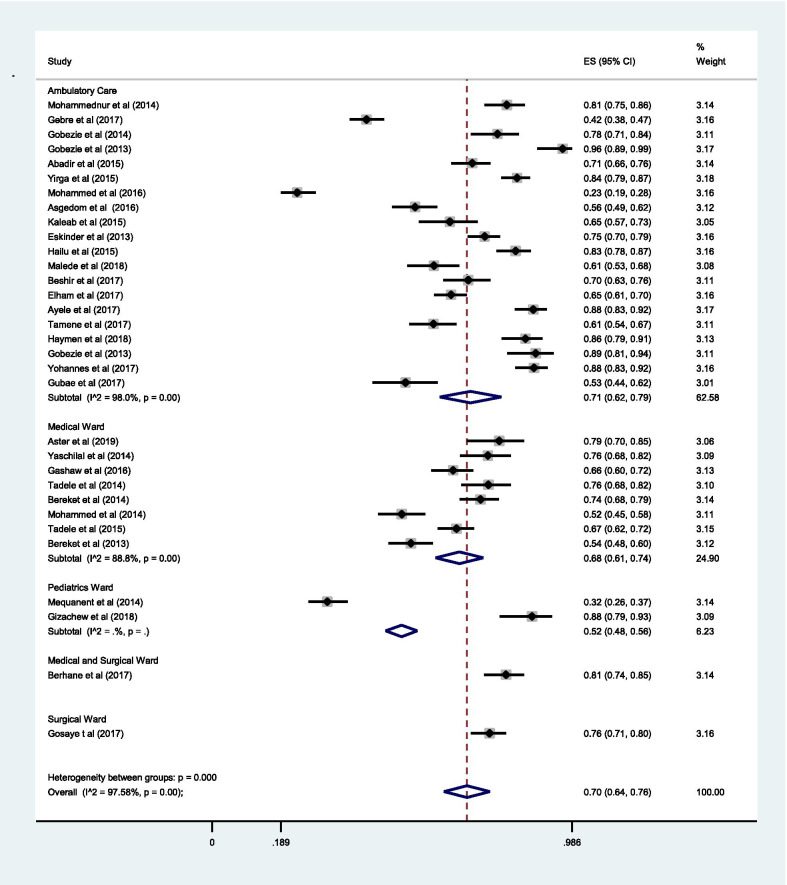
Sub-group analysis of drug-related problems by the department of the hospital in which the study conducted

The sub-group analysis by the departments of the hospital indicated, the estimated pooled prevalence of DRPs at ambulatory care [71% (0.71; 95% CI 0.62–0.79; *I*^2^ = 98.0% *p* = 0.00)] was slightly higher as compared to the estimated pooled prevalence at the medical ward [68% (0.68; 95% CI 0.61–0.74; *I*^2^ = 88.8% *p* = 0.00)] (Fig. 11).

**Fig. 12:**
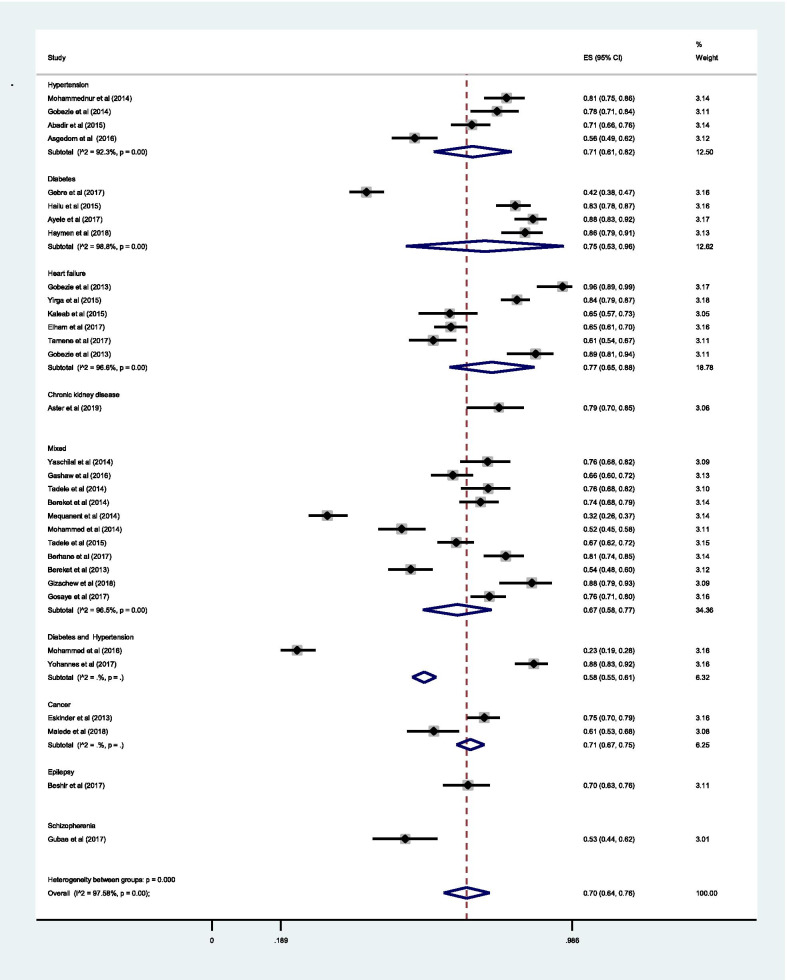
Sub-group analysis of drug-related problems by medical conditions

The sub-group analysis by medical condition showed that the estimated pooled prevalence of DRPs among hypertensive patients [71% (0.71;95% CI 0.61–0.82; *I*^2^ = 92.32 *p* = 0.00)] was slightly lower compared to the pooled estimate among diabetic [75% (0.75; 95% CI 0.53–0.96; *I*^2^ = 98.76% *p* = 0.00)] and heart failure patients [77% (0.77; 95% CI 0.65–0.88; *I*^2^ = 96.62% *p* = 0.00)]; but slightly higher compared to pooled estimate among patients with more than one medical conditions (i.e., unspecified cases from medical, surgical and pediatrics ward) [67% (0.67; 95% CI0.58–0.77; *I*^2^ = 96.53% *p* = 0.00)] (Fig. 12).

### Factors associated with DRPs

The pooled estimate of factors associated with the occurrence of DRPs indicated that, patients who receive polypharmacy had 1.3 times more chance to experience DRPs as compared to those who were not on polypharmacy [(RR = 1.3:95% CI 1.187–1.43)]. Similarly, patients who had a comorbid medical condition had 1.293 times more likely to encounter DRPs compared to patients with no medical comorbidity [(RR = 1.3; 95% CI 1.16–1.44)]. Other factors such as having type 2 diabetes [(RR = 1.8; 95% CI1.08—2.98)], poor medication adherence [(RR = 1.7; 95% CI 1.28—2.39)], uncontrolled blood pressure [(RR = 1.4;95% CI1.28–2.39)], substance use [(RR = 1.2; 95%CI1.06–1.38)], significant drug interactions [(RR = 1.33; 95% CI1.05–1.69)], a negative belief about medicine [(RR = 3.72; 95% CI 2.31—5.97)], talking ≥ 3 drugs [(RR = 1.47; 95% CI 1.22—1.77)], were also significantly associated with the occurrence of DRPs (Table 3).

**Table 3:** Factors associated with the occurrence of DRPs in Ethiopia

**Factors**	**RR (95% CI)**	***Z-statistics***	***p-value***
Comorbidity	1.3 (1.16–1.44)	4.73	*p* = 0.000
Taking ≥ 5 drugs	1.3 (1.187–1.43)	5.59	*p* = 0.000
Type 2 diabetes	1.8 (1.08–2.98)	2.24	*p* = 0.025
Poor medication adherence	1.7 (1.28–2.39)	3.49	*p* = 0.000
Uncontrolled BP	1.4 (1.09–1.84)	2.56	*p* = 0.010
Negative medication belief	3.72 ( 2.31–5.97)	5.43	*p* = 0.000
Significant drug interaction	1.33 ( 1.05–1.69)	2.33	*p* = 0.020
Talking ≥ 3 drugs	1.47 (1.22–1.77)	4.07	*p* = 0.000
Substance use	1.2 ( 1.06–1.38)	2.87	*p* = 0.004
Taking ≥ 2 drugs	1.3 (1.04–1.59)	2.27	*p* = 0.023

*RR* Relative Risk, *BP* Blood Pressure

### Publication bias

**Fig. 13:**
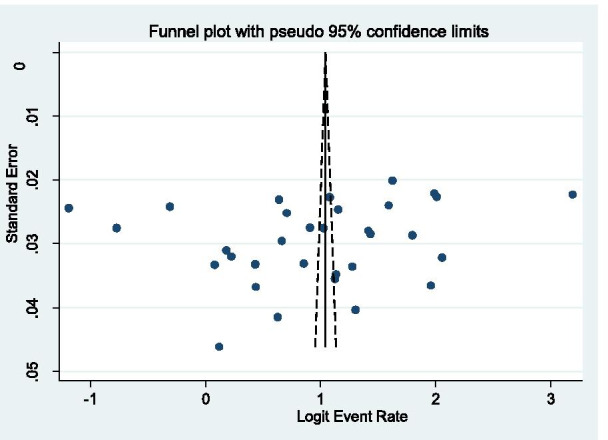
Funnel plot of logit event by the standard error of event for publication bias

A test for publication bias was performed using Begg’s correlation and Egger’s regression test. Both tests did not show evidence for the presence of publication bias among the included studies (*p* = 0.178 and *p* = 0.213, respectively) (Fig. 13).

## Discussion

To the best of our knowledge, this is the first systematic review and meta-analysis to determine the prevalence of DRPs and associated factors in Ethiopia. This review included 32 studies that evaluated the prevalence and factors associated with the occurrence of DRPs. Of the total included studies, the majority (*n* = 20) were conducted in the ambulatory care settings where heart failure, hypertension, and diabetes were the most commonly involved chronic medical conditions.

The pooled prevalence estimate of DRPs in this review was comparable with the prevalence of DRPs reported in the study conducted in Minnesota [11]. However, it was lower than the prevalence identified in studies conducted in Malaysia (90.5% [10], Brazil (91.7%) [56] and Kenya (93.8%) [57]; but higher than the result reported in the studies conducted in Nigeria [58], Saudi Arabia (45.2%) [59], China (21.0%) [13] and Spain (29.8%) [12].

The sub-group analysis by the hospital department showed that the magnitude of DRPs was slightly higher at the ambulatory care compared to the medical wards. This could perhaps due to the fact that patents treated in the ambulatory care settings were slightly older as as compared to other settings, and often with multiple medical co-morbidities, taking multiple drugs and a complex regimen potentially predisposing them to experience more DRPs. Besides, a sub-group analysis by a medical condition showed that the magnitude of DRPs among heart failure patients was higher than hypertensive and diabetic patients. This may be due to the fact that patients with heart failure had more co-morbidities and multiple medications and thus, may have experienced higher rates of DRPs compared to patients with diabetes and hypertension [60]. The estimated magnitude of DRPs in patients with hypertensive and diabetes in this review was higher compared to the magnitude reported in a study conducted in Nigeria; 49.8% and 50.2% in hypertensive and diabetic patients, respectively [58]. Likewise, the estimated magnitude of DRPs in heart failure patients was higher compared to results reported in a study conducted in Spain (29.8%) [58] and North Cyprus (63%) [61].

The finding from the pooled estimate of factors associated with DRPs revealed that the number of drugs used was significantly influenced the risk to experience DRPs. This result was similar to the finding from a study conducted in China [13] and Spain [12]. Taking multiple drugs has been linked to adverse health outcomes including drug interactions and poor adherence to treatment. Moreover, our review showed that presence of medical comorbidities was significantly associated with the occurrence of DRPs. This could imply that patients with medical comorbidity often use multiple medications that predispose them to one or more DRPs. Likewise, drug interactions were significantly associated with the occurrence of DRPs in this review. Drug interactions can lead to an increase or a decrease in the clinical effect of one or more of combined drugs which predisposes the patient to encounter DRPs. Our review also found that uncontrolled blood pressure was significantly associated with occurrence of DRPs. Patients with uncontrolled blood pressure often receive combination of multiple drugs and the use of concurrent multiple medicines might be a factor predisposing patients to DRPs. Likewise, in our review, poor medication adherence and a negative medication belief were significantly associated with the occurrence of DRPs. Poor medication adherence, and having a negative belief about medicine are often associated with compliance- related DRPs and this was found as the most common sub-types of DRPs in our review. Furthermore, substance use was significantly associated with the occurrence of DRPs in this review. This might be due to the fact that self medication with various substances may result in drug drug-drug/drug-substance interactions or patients not taking their medications appropriately.

The pooled estimate of the proportion of DRPs by sub-types indicated that the indication related problems (need additional drug therapy) were the most frequently encountered DRPs. This reflects that most patients require initiation of additional drug therapy for their untreated medical condition or prophylactic purpose. The magnitude of indication related problems (need additional drug therapy) identified in this review was lower than the magnitude observed in studies conducted in Kenya (39.2%) [62] and Nigeria (100%) [63], but higher than the magnitude reported in studies conducted in Spain (3.13%) [58] and North Cyprus (20%) [64]. The proportion of indication related problems (unnecessary drug therapy) was slightly lower compared to the proportion reported in a study done in Kenya (12.4%) [57], but higher than the proportion reported in a study conducted in China (7.3%) [13]. The inconsistency could be due to the difference in the categorization of DRPs, sample size, and experience in PC practice in the institutions. On the other hand, effectiveness related problems (dose too low) were the second most frequently encountered DRPs sub-types; indicating that patients were using sub-optimal therapeutic doses which did not result in a desired clinical response. Compared to our study, a higher magnitude of effectiveness related problems (dose too low) was reported in the study undertaken in Brazil (20.8%) [63] and Kenya (19.8%) [63], but the lower figure was reported in the study done in Jagdalpur (7.63%) [17] and Malaysia (1.3%) [10]. Another effectiveness related problems (ineffective drug) in this review was higher than the effectiveness related problems identified in the study done in Brazil (9.2%) [56] and Malaysia (8.8%) [10], but it was lower than the effectiveness related problems reported in the study performed in Nigeria (28.4%) [63]. This discrepancy could be explained by a difference in the classification of drug-related problems, the experience in PC service, study design, and sample size.

The proportion of safety-related problems (adverse drug reaction) in this review was comparable to the proportion identified in the study done in China (11.0%) [13] but lower than the proportion reported in the study conducted in Nigeria (40.4%) [63]. The proportion of another safety-related problem (dose too high) was lower than the proportion reported in the study done in Malaysia (11.3%) [10] and China (15.9%) [13], but higher than the proportion reported in the study undertaken in Brazil (1.6%) [56]. This variation could be due to the difference in study design, categorization of drug-related problems, and experience in a practice setting.

The proportion of non-compliance in this review was lower than the magnitude identified in the study performed in Jagdalpur (46.6%) [17], Kenya (32.1%) [57], South-West Nigeria (55.4%) [58], Brazil(25.0%) [56]; however, it was higher than the proportion of non-compliance stated in the study done in Malaysia (12.9%) and Brazil (4.7%) [56]. This inconsistency might be due to differences in the study population, categorization of DRPs, and PC practice of healthcare professionals in the setting.

### Strength and limitation

This systematic review and meta-analysis were the first of its kind to estimate and pointed out the prevalence, sub-types, and factors associated with DRPs in Ethiopia. However, it was not without limitations. Some of the included studies differ in their design, types of patients involved, medical conditions and medications used, and health care settings in which the studies were conducted. This has limited our ability to draw conclusions on some data such as typs/class of medication most commonly contributing to occurrence of DRPs and priority areas for prevention of DRPs in resource limited settings.

## Conclusion

The estimated national prevalence of DRPs in Ethiopia was seemed high. The magnitude of DRPs was slightly higher at ambulatory care and among patients with hypertension, heart failure, and diabetes. Moreover, need additional drug therapy, low dose, and non-compliance were among the frequently encountered DRP sub-types. The number of drugs used, significant drug interaction, poor medication adherence, uncontrolled blood pressure, type 2 diabetes, substance use, a negative medication belief, and medical comorbidity were the factors that significantly influenced the occurrence of DRPs. Improving involvements of clinical pharmacist in the multidisciplinary health care team, and initiating or and strengthening the pharmaceutical care service at every health care facility in the country should be considered, since clinical pharmacist has a significant contribution in identification and resolution of DRPs.

## Supplementary Information

**Additional file 1.** Raw data.

## Data Availability

The raw data used in the review was submitted as Additional file 1.

## References

[1] Wiedenmayer KRSSC. Developing pharmacy practice A focus on patient care Handbook – 2006 edition. 2006. 87 p. http://www.fip.org/files/fip/publications/DevelopingPharmacyPractice/DevelopingPharmacyPracticeEN.pdf

[2] Pickard AS, Johnson JA, Farris KB (1999). Defining clinical pharmacy: a new paradigm. Ann Pharmacother.

[3] Pedley KM, Advisor CI, Resources H. HRSA ’ s Patient Safety and Clinical Pharmacy Services Collaborative What is the Collaborative ? 2007.

[4] Luisetto M, Sahu RK (2016). Clinical Pharmaceutical Care: A New Management Health Care Discipline. J Pharm Biosci.

[5] APH. Principles of practice for pharmaceutical care. 2012. http://www.pharmacist.com/principles-practice-pharmaceutical-care

[6] Youssef A (2004). Pharmaceutical Care Practice: The Clinician’s Guide. Int J Toxicol..

[7] ASHP (1996). ASHP guidelines on a standardized method for pharmaceutical care. Am J Heal Pharm..

[8] Blix HS, Hospital LD. Drug-related problems in hospitalised patients: a prospective bedside study of an issue needing. 2007.

[9] Gayathri B, Gayathri B, Divasish LE, Soni M, Hup GK, Prasath KH (2018). Drug related problems: a systemic literature review. Int J Pharm Ther.

[10] Zaman Huri H, Fun Wee H (2013). Drug related problems in type 2 diabetes patients with hypertension: a cross-sectional retrospective study. BMC Endocr Disord..

[11] Rao D, Gilbert A, Strand LM, Cipolle RJ (2007). Drug therapy problems found in ambulatory patient populations in Minnesota and South Australia. Pharm World Sci.

[12] Urbina O, Ferrández O, Luque S, Grau S, Mojal S, Pellicer R (2014). Patient risk factors for developing a drug-related problem in a cardiology ward. Ther Clin Risk Manag.

[13] Rashed AN, Wilton L, Lo CCH, Kwong BYS, Leung S, Wong ICK (2014). Epidemiology and potential risk factors of drug-related problems in Hong Kong paediatric wards. Br J Clin Pharmacol.

[14] Bedouch P, Allenet B, Grass A, Labarère J, Brudieu E, Bosson JL (2015). Drug-related problems in medical wards with a computerized physician order entry system. Clin Pharm Theraoeutics..

[15] Koh Y, Kutty FBM, Li SC (2005). Drug-related problems in hospitalized patients on polypharmacy: the influence of age and gender. Ther Clin Risk Manag.

[16] Maingi AW. Assessment of drug therapy problems in adult patients with both cardiovascular diseases and type 2 diabetes mellitus at Kenyatta National Hospital. 2018.

[17] Singh H, Kumar BN, Sinha T, Dulhani N (2011). The incidence and nature of drug-related hospital admission: a 6-month observational study in a tertiary health care hospital. J Pharmacol Pharmacother.

[18] Ernst FR, Grizzle AJ (2001). Drug-related morbidity and mortality: Updating the cost-of-illness model. J Am Pharm Assoc..

[19] Johnson JA, Boatman LI (1996). Drug-Related Morbidity and Mortality : A Cost-of-Illness Model. J Manag Care Pharm..

[20] Linda M. Strand, Robert J. Cipolle PCM and MJF. The impact of pharmaceutical care practice on the practitioner and the patient in the ambulatory practice setting: Twenty-five years of experience. Curr Pharm Des. 2004;10(31):3987–4001. http://ovidsp.ovid.com/ovidweb.cgi?T=JS&PAGE=reference&D=emed6&NEWS=N&AN=2004520026 10.2174/1381612043382576 15579084

[21] Education P (2016). Journal of clinical & experimental the position of clinical pharmacists in delivering advanced pharmacy practice education and services: short communication. Clin Exp Pharmacol.

[22] The Joanna Briggs Institute (JBI). The Joanna Briggs Institute Critical Appraisal tools for use in JBI Systematic Reviews. Critical Appraisal Checklist for Analytical Cross Sectional Studies. 2017.

[23] The Joanna Briggs Institute (JBI). The Joanna Briggs Institute Critical Appraisal tools for use in JBI Systematic Reviews. Critical Appraisal Checklist for Cohort Studies. 2017.

[24] Temesgen G, Kefale B, Degu A (2014). Drug Therapy Problem among patients with Cardiovascular Diseases in Felege Hiwot Referral Hospital, North East, Bahir-Dar ethiopia. Indo Am J Pharm Res..

[25] Seid E (2018). Assessment of drug therapy problems among ambulatory heart failure patients at Tikur Anbessa Specialized Hospital.

[26] Garedow AW, Bobasa EM, Wolide AD, Dibaba FK, Fufa FG, Tufa BI, et al. Drug-related problems and associated factors among patients admitted with chronic kidney disease at Southwest Ethiopia : a hospital-based prospective observational study. Int J Nephrol. 2019. 10.1155/2019/1504371 PMC6854244 31772774

[27] Legesse Y, Id N, Kumela K, Kassa TD, Angamo T (2018). Drug therapy problems and contributing factors in the management of heart failure patients in Jimma University Specialized Hospital Southwest Ethiopia. PLoS ONE.

[28] Yadesa TM, Gudina EK, Angamo MT (2015). Antimicrobial use-related problems and predictors among hospitalized medical in- patients in southwest Ethiopia: prospective observational study. PLoS ONE.

[29] Tigabu BM, Daba D, Habte B (2014). Journal of Research in Pharmacy Practice Drug - related problems among medical ward patients in Jimma university specialized hospital, Southwest Ethiopia. J Res Pharm Pract.

[30] Yimama M, Jarso H, Desse TA (2018). Determinants of drug - related problems among ambulatory type 2 diabetes patients with hypertension comorbidity in Southwest Ethiopia : a prospective cross sectional study. BMC Res Notes..

[31] Hailu BY, Berhe DF, Gudina EK, Gidey K, Getachew M (2020). Drug related problems in admitted geriatric patients : the impact of clinical pharmacist interventions. BMC Geriatr..

[32] Tigabu BM, Daba D, Habte B (2013). Factors associated with unnecessary drug therapy and inappropriate dosage in hospitalized patients in Jimma University Specialized Hospital, South West Ethiopia. World J Pharm Sci..

[33] Tefera GM, Zeleke AZ, Jima YM, Kebede TM (2020). Drug therapy problems and the role of clinical pharmacist in surgery ward: prospective observational and interventional study. Drug Healthc Patient Saf.

[34] Teklemariam G, Id D, Beyene A, Id B, Woldu MA (2019). Drug therapy problems, medication adherence and treatment satisfaction among diabetic patients on follow-up care at Tikur Anbessa Specialized Hospital. Addis Ababa, PLoS ONE.

[35] Sisay EA, Engidawork E, Yesuf TA, Ketema EB. Drug Related Problems in Chemotherapy of Cancer Patients. J Cancer Sci Ther. 2015;7: 2.

[36] Berihun M (2019). Identification and Resolution of Drug Related Problems in Pediatric Hematology/Oncology Ward of Tikur Anbessa Specialized Hospital.

[37] Bedru B. Assessment of Drug Therapy Problems among Ambulatory Epileptic Patients at Tikur Anbessa Specialized Hospital ,. 2018;

[38] Biset M. Assessment of Drug Related Problems in Medical Wards of Tikur Anbessa Specialized Hospital. 2015;(January). 10.4103/2279-042X.167048 PMC4645135 26645029

[39] Temtem G, Submitted T, Pharmacy C (2018). Assessment of Drug Therapy Problems and Associated Factors Among Ambulatory Patients with Schizophrenia at Tikur Anbessa Specialized Hospital, Addis Ababa.

[40] Abdulmalik H, Tadiwos Y, Legese N. Assessment of drug ‑ related problems among type 2 diabetic patients on follow up at Hiwot Fana Specialized University. BMC Res Notes [Internet]. 2019;4–9. Available from: https://doi.org/10.1186/s13104-019-4760-8 10.1186/s13104-019-4760-8 PMC6880367 31771634

[41] Ayele Y, Melaku K, Dechasa M, Ayalew MB, Horsa BA. Assessment of drug related problems among type 2 diabetes mellitus patients with hypertension in Hiwot Fana Specialized University Hospital , Harar , Eastern Ethiopia. BMC Res Notes [Internet]. 2018;11(:728):1–5. Available from: https://doi.org/10.1186/s13104-018-3838-z 10.1186/s13104-018-3838-z PMC6186051 30314443

[42] Gelchu T, Abdela J (2019). Drug therapy problems among patients with cardiovascular disease admitted to the medical ward and had a follow-up at the ambulatory clinic of Hiwot Fana Specialized University Hospital : The case of a tertiary hospital in eastern Ethiopia. SAGE Open Med.

[43] Belayneh YM, Amberbir G, Agalu A (2018). A prospective observational study of drug therapy problems in medical ward of a referral hospital in northeast Ethiopia. BMC Health Serv Res..

[44] Bizuneh GK, Adamu BA, Bizuayehu GT, Adane SD (2020). A Prospective Observational Study of Drug Therapy Problems in Pediatric Ward of a Referral Hospital.

[45] Hussein M, Lenjisa JL, Woldu MA, Tegegne GT, Umeta GT, Dins H (2014). Assessment of drug related problems among hypertensive patients on follow up in Adama Hospital Medical College. East Ethiopia Clin Pharmacol Biopharm.

[46] Tegegne GT, Gaddisa T, Kefale B, Tesfaye G, Likisa J, Albachew M (2015). Drug therapy problem and contributing factors among ambulatory hypertensive patients in Ambo General Hospital, West Shoa. Ethiopia. Glob J.

[47] Abadir Hussen FBD (2017). Drug Therapy Problems and their predictors among hypertensive patients on follow up in Dil-chora Referral Hospital, Dire-Dawa Ethiopia. IJPSR.

[48] Weldegebreal AS, Tezeta F, Mehari AT, Gashaw W (2019). Assessment of drug therapy problem and associated factors among adult hypertensive patients at Ayder comprehensive specialized hospital Northern Ethiopia. Afr Health Sci.

[49] Gizaw K, Dubale M (2017). Drug Related Problems and Contributing Factors Among Adult Ambulatory Patients with Cardiovascular Diseases at Gebretsadik. J Nat Res.

[50] Koyra HC, Tuka SB, Tufa EG (2017). Epidemiology and predictors of drug therapy problems among type 2 diabetic patients at Wolaita Soddo University Teaching Hospital Southern Ethiopia. Am J Pharmacol Sci.

[51] Argaw AM, Hiwet TT, Derse BB (2019). Drug Therapy Problems and Determinants among Ambulatory Type 2 Diabetes Mellitus Patients : Pharmacists ’ Intervention in South-East Endocrinology & Metabolic Syndrome. Endocrinol Metab Syndr.

[52] Tegegne GT, Yimamm B, Yesuf EA (2015). Drug Therapy Problems & Contributing Factors Among Patients with Cardiovascular Diseases in Felege Hiwot Referral and Jimma University Specialized Hospital Ethiopia. INDO Glob J Pharm Sci.

[53] Yadesa TM (2017). Inappropriate Use of Antimicrobials and the Determinants among Patients Hospitalized in 3 Hospitals ( Mizan, Bonga and Tepi ) in Southwest Ethiopia. J Bioanal Biomed..

[54] Meknonnen GB, Biarra MK, Tekle MT, Bhagavathula AS (2017). Assessment of Drug Related Problems and its Associated Factors among Medical Ward Patients in University of Gondar Teaching Hospital, Northwest Ethiopia : A Prospective Cross-Sectional Study. J Basic Clin Pharma.

[55] Birarra MK, Bacha T, Heye WS (2018). Assessment of drug-related problems in pediatric ward of Zewditu Memorial Referral Hospital, Addis Ababa Ethiopia. J Clin Pharm..

[56] Nascimento YDA, Carvalho WDS, Acurcio FDA (2009). Drug-related problems observed in a pharmaceutical care service, Belo Horizonte Brazil. Braz J Pharm Sci.

[57] Degu A, Njogu P, Weru I, Karimi P (2017). Assessment of drug therapy problems among patients with cervical cancer at Kenyatta National Hospital. Kenya BioMed Cent.

[58] Adisa R, B-f DOO (2019). Evaluation of drug therapy problems among outpatient hypertensive and type-2-diabetic patients at a tertiary hospital. South-West Nigeria Nig J Pharm Res.

[59] Ibrahim N, Wong IC, Patey S, Tomlin S, Sinha MD, Jani Y (2013). Drug-related problem in children with chronic kidney disease. Pediatr Nephrol.

[60] Yancy CW, Jessup M, Bozkurt B, Butler J, Casey DE, Colvin MM (2017). 2017 ACC/AHA/HFSA Focused Update of the 2013 ACCF/AHA Guideline for the Management of Heart Failure: a Report of the American College of Cardiology/American Heart Association Task Force on Clinical Practice Guidelines and the Heart Failure Society of America. Circulation.

[61] Gökçekuş L, Mestrovic A, Basgut B (2016). Pharmacist intervention in drug-related problems for patients with cardiovascular diseases in selected community pharmacies in Northern Cyprus. Trop J Pharm Res.

[62] Blix HS, Viktil KK, Moger TA, Reikvam Å (2006). Characteristics of drug-related problems discussed by hospital pharmacists in multidisciplinary teams. Pharm World Sci.

[63] Samaila A, Biambo AA, Usman N, Aliyu HH (2019). Drug related problems and implications for pharmaceutical care interventions in hypertensive outpatients in a Nigerian hospital Drug related problems and implications for pharmaceutical care interventions in hypertensive outpatients in a Nigerian hospital. J Sci Pract Pharm.

[64] Kaufmann CP, Stämpfli D, Hersberger KE, Lampert ML (2015). Determination of risk factors for drug-related problems: a multidisciplinary triangulation process. BMJ Open.

